# A Relationship Prediction Method for *Magnaporthe oryzae*–Rice Multi-Omics Data Based on WGCNA and Graph Autoencoder

**DOI:** 10.3390/jof9101007

**Published:** 2023-10-12

**Authors:** Enshuang Zhao, Liyan Dong, Hengyi Zhao, Hao Zhang, Tianyue Zhang, Shuai Yuan, Jiao Jiao, Kang Chen, Jianhua Sheng, Hongbo Yang, Pengyu Wang, Guihua Li, Qingming Qin

**Affiliations:** 1 College of Computer Science and Technology, Jilin University, Changchun 130012, China; zhaoes22@mails.jlu.edu.cn (E.Z.); dongly@jlu.edu.cn (L.D.); zg351047967@163.com (H.Z.); tianyue20@mails.jlu.edu.cn (T.Z.); jiaojiao21@mails.jlu.edu.cn (J.J.); chenkang21@mails.jlu.edu.cn (K.C.); shengjh21@mails.jlu.edu.cn (J.S.); 2 Key Laboratory of Symbolic Computation and Knowledge Engineering of Ministry of Education, Jilin University, Changchun 130012, China; 3 College of Software, Jilin University, Changchun 130012, China; yuanshuai20@mails.jlu.edu.cn (S.Y.); yanghb21@mails.jlu.edu.cn (H.Y.); wangpy22@mails.jlu.edu.cn (P.W.); 4 College of Plant Science, Key Laboratory of Zoonosis Research, Ministry of Education, Jilin University, Changchun 130012, China; liguihua@jlu.edu.cn; 5 Department of Molecular Microbiology and Immunology, School of Medicine, University of Missouri, Columbia, MI 65211-7310, USA; qingmingqin@yahoo.com

**Keywords:** cross-kingdom regulation, *Magnaporthe oryzae Oryzae* (MoO) pathotype, rice, multi-omics, WGCNA, graph autoencoder

## Abstract

*Magnaporthe oryzae Oryzae* (MoO) pathotype is a devastating fungal pathogen of rice; however, its pathogenic mechanism remains poorly understood. The current research is primarily focused on single-omics data, which is insufficient to capture the complex cross-kingdom regulatory interactions between MoO and rice. To address this limitation, we proposed a novel method called Weighted Gene Autoencoder Multi-Omics Relationship Prediction (WGAEMRP), which combines weighted gene co-expression network analysis (WGCNA) and graph autoencoder to predict the relationship between MoO–rice multi-omics data. We applied WGAEMRP to construct a MoO–rice multi-omics heterogeneous interaction network, which identified 18 MoO small RNAs (sRNAs), 17 rice genes, 26 rice mRNAs, and 28 rice proteins among the key biomolecules. Most of the mined functional modules and enriched pathways were related to gene expression, protein composition, transportation, and metabolic processes, reflecting the infection mechanism of MoO. Compared to previous studies, WGAEMRP significantly improves the efficiency and accuracy of multi-omics data integration and analysis. This approach lays out a solid data foundation for studying the biological process of MoO infecting rice, refining the regulatory network of pathogenic markers, and providing new insights for developing disease-resistant rice varieties.

## 1. Introduction

Rice (*Oryza sativa* L.) is one of the most important staple foods worldwide, playing a crucial role in people’s daily diet and clothing. Rice production not only directly affects global food security but also contributes significantly to the development of industries and economies. However, rice diseases, especially rice blast caused by the fungus *Magnaporthe oryzae *Oryzae** (MoO) pathotype, can lead to significant yield losses [1]. Rice blast is the most devastating disease in rice, resulting in 10–20% yield reduction generally and up to 50% losses in severe outbreaks. Moreover, different pathotypes of *M. oryzae* can also infect other cereal crops like barley, wheat, sorghum, and corn, posing a serious threat to global food security [2,3]. Therefore, uncovering the molecular mechanism of MoO infection is imperative for devising sustainable solutions against rice blast.

MoO is an excellent model fungus for studying pathogen–plant interactions. However, its high mutation rate leads to the breakdown of rice blast resistance within a few years [4]. Chemical control methods are expensive and difficult to apply on a large scale [5]. To develop broad-spectrum and durable solutions, researchers have conducted extensive research work on the MoO–rice pathosystem using various omics approaches. However, elucidating the intricate host–pathogen crosstalk solely through biological experiments remains challenging [6,7,8,9]. The advent of various omics datasets on MoO–rice interactions provides new opportunities to uncover key regulators through computational approaches. While multi-omics data integration methods have shown promising results in human complex disease prognosis and classification [10,11,12,13,14], the research on fungal infection in plants using these methods has only recently begun [15,16,17]. Significant experimental data on MoO–rice interactions have been generated, but analyzing such complex, heterogeneous, and often imbalanced datasets to reveal novel biological insights remains an unmet challenge.

Regarding experimental data, the whole genome sequencing of MoO and rice has been completed [18,19,20,21], and 183 rice blast resistance genes have been identified [22]. However, rice blast resistance genes obtained through genetic improvement often come at the cost of yield reduction, and newly bred rice varieties may lose resistance to the disease after several years [23,24]. In the transcriptome, a considerable amount of data related to the interaction between MoO and rice have been stored in databases like Gene Expression Omnibus (GEO) [25]. Studies have revealed that some fungal pathogens, such as *Botrytis cinerea* [26,27], *Blumeria graminis* [28], and *Rhizoctonia solani* [29], use sRNAs to silence host immune genes and facilitate infections by these pathogens. Nair et al. [30] reported that the plant immune system is mainly composed of two layers of immune responses: Pattern-Triggered Immunity (PTI) and Effector-Triggered Immunity (ETI). PTI is mainly triggered by pathogen-related molecular patterns on the surface of pathogenic microorganisms, and ETI is triggered by the plant’s disease-resistant proteins recognizing the effector proteins produced by pathogenic microorganisms. At the same time, gene regulation mediated by noncoding RNAs are also involved in plant defense responses. Raman et al. [31] demonstrated that the loss of a single gene encoding Dicer, RNA-dependent RNA polymerase, or Argonaute, which are required for the biogenesis of sRNA-matching genome-wide regions (coding regions, repeats, and intergenic regions), reduces the sRNA levels in *M. oryzae*. The loss of one Argonaute reduced both the sRNA and fungal virulence on barley leaves. A transcriptome analysis revealed that sRNA play an important role in the transcriptional regulation of repeats and intergenic regions in *M. oryzae*. These data support that *M. oryzae* sRNAs play an important regulatory role in fungal development and virulence. In the proteome, 45 pathogenic proteins linked to rice blast have been cataloged in the PHI-base database [32,33], and some fungal proteins [34,35,36] and plant proteins [37,38] have been implicated in the process of the fungal infection of plants. Although the proteome data of MoO and rice are relatively abundant, some crucial proteins are expressed in low abundance, and many unknown functional proteins may play a vital role in infection [39].

To study the molecular mechanisms of fungi–plant interactions and biological pathways related to resistance, researchers have adopted multi-omics research methods to perform correlation analyses on omics data like plant transcriptome, proteome, and metabolome [23,40,41,42]. Meanwhile, the methods and tools for integrating multi-omics data are expanding. In data integration, researchers often employ deep learning methods to uncover the key driving factors and expand the association networks in various omics data [43,44]. Multi-omics analysis algorithms like 3Omics [45], BiofOmics [46], iCluster [47], and SNF [48] have yielded promising results in bioinformatics.

Existing studies on genomes have combined plant disease resistance genes and sRNAs for analysis [49]. However, while some sRNAs [50,51], proteins [34,35,36,37,38], and metabolites [52] have been identified through other omics, it is still challenging to fully present the complex biomolecular interaction process of MoO infection at different omics levels from a single omics. Combining multi-omics data can effectively mine the relationship between groups of biomolecules. The key challenge is how to analyze the heterogeneous network composed of the relationship data between different omics of MoO infecting rice to fully present the complex biomolecular interaction process in the entire infection process. Moreover, the infection of rice by MoO involves transboundary regulation [50,53]. The challenge of cross-kingdom regulation lies in the difficulty in verifying the biological process through direct biological experiments [54]. Thus, we can only indirectly verify the relationship through data model prediction. Furthermore, it is difficult to accurately extract the mixed data obtained after MoO infecting rice into pure MoO or rice data, then conduct a differential expression analysis with the data before infection. At the same time, data imbalance in multi-omics data during MoO infecting rice is also a problem. Unfortunately, there is no method for analyzing biological processes of transboundary regulation. Therefore, there is an urgent need to develop a multi-omics heterogeneous data prediction algorithm for cross-kingdom regulation to solve this problem.

In this work, we propose a novel algorithm, Weighted Gene Autoencoder Multi-Omics Relationship Prediction (WGAEMRP), an integrated weighted gene co-expression network analysis (WGCNA) and graph autoencoders, to predict unknown relationships from multi-omics data on MoO–rice interactions. We constructed an integrative network from genome, transcriptome, sRNA, proteome, and metabolome data and discovered the MoO sRNAs that play a key role in the infection of rice by the fungal pathogen. Our approach provides an effective solution to mine cross-kingdom relationships from the heterogeneous network and addresses data imbalance issues. The predicted network expands our understanding of rice blast infection mechanisms and identifies potential sRNA regulators and biomarkers that may play important regulatory roles in MoO–rice interactions. This study provides analytical tools to comprehensively elucidate MoO–rice interactions and guide disease management strategies.

## 2. Materials and Methods

### 2.1. Data Resources and Preprocessing

The data used in this study include whole genome data of MoO and rice based on 16 h of MoO gene expression data on a complete culture medium, gene expression data of a MoO and rice mixture obtained 72 h after MoO infecting rice, gene expression data of rice cultured for 48 h, rice gene expression data 48 h after infection with MoO, mRNA data of MoO and rice, model pathogen–model host protein interaction data, and protein sequence data for model pathogens and model hosts, as well as protein sequence data for MoO and rice. The National Center for Biotechnology Information (NCBI), Universal Protein Resource (UniProt), and Host–Pathogen Interaction (HPIDB) databases were used as data sources.

First, quality control and data filtering were performed on the gene expression data of MoO and rice in order to obtain high-quality data. Secondly, bowtie, hisat2, and samtools were used to map the post-infection data to the genomes of MoO and rice to preliminarily classify which ones belong to MoO and which belong to rice. Next, we used the R language Subreads package, edgeR package, and reshape2 package to calculate the gene expression and established the rice expression matrix before and after MoO infection.

Then, we performed clustering screening on the rice genome data through WGCNA to find the rice genes that are closely related to rice blast expression. We mainly conducted a relevant analysis on rice data through the expression matrix constructed from rice genome data. We then clustered rice genes with similar expression patterns into the same module and analyzed the correlation between the module and disease traits, as well as the correlation coefficient between the module and the sample, to select the module most relevant to the disease. Next, we analyzed the module and found the key genes in the module.

Since the length of a miRNA (a type of sRNA that inhibits gene expression) is between 18 and 25 nt, it is believed that the length of MoO sRNAs that can target rice genes for cross-kingdom regulation should also be within this range. After length control, count the number of occurrences of each sequence (i.e., expression amount). Then, match file A that has not been genome mapped (containing the sRNA sequence, sequence length, and sequence expression) with file B that has been genome mapped (contains only deduplicated sequences). The sequences in file A that do not appear in file B are discarded. This specific method is to sort the expression levels of MoO sRNA from high to low, find the MoO sRNA ranked at 3/4, and use this expression level as the baseline for lower expression levels. The expression levels of other samples are converted to multiples of this expression level. The standardized differentially expressed sRNA data of MoO were statistically analyzed, and the differentially expressed MoO sRNAs were screened based on the expression amount and rate.

Then, the protein interaction prediction method based on sequence features (Interolog method) is based on the principle that homologous proteins have similar functional structural characteristics. The protein interaction prediction method based on functional domains (Domain–Domain method) is based on the principle that interacting protein pairs may have the same functional domain. Based on the experimentally verified protein interaction relationship between the model pathogen and the model host, we used the Interolog method and the Domain–Domain method and screened the MoO-secreted proteins through TMHMM to predict the MoO–rice protein interaction pair. Finally, we obtained the relationship data between the MoO fungus and rice multi-omics using the omics data obtained above.

### 2.2. Algorithm Ideas

**Figure 1:**
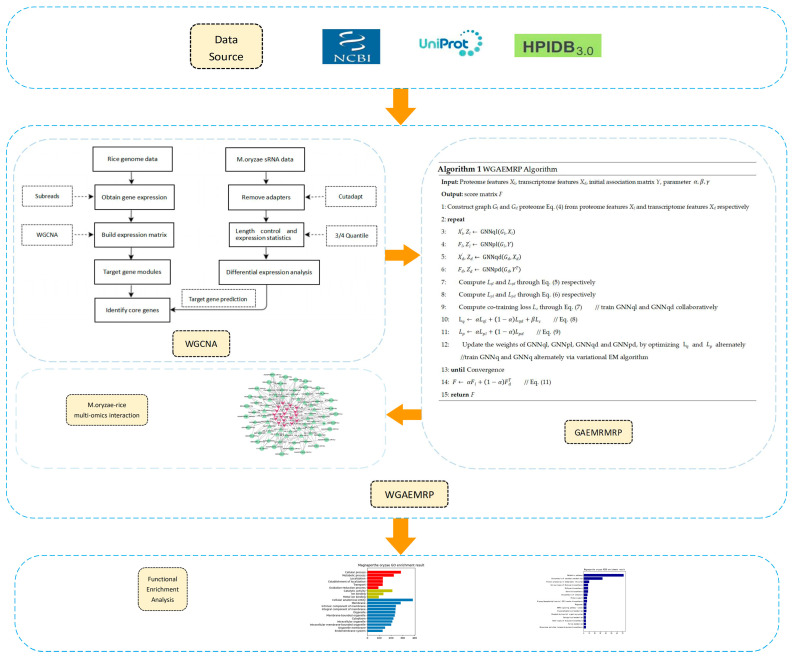
Overall flow chart of this work. First, we downloaded the required multi-omics data from the data source. Next, we applied the WGCNA method and some software packages to obtain the known interaction relationships between MoO–rice omics key factors. Then, we applied the GAEMRMRP algorithm to predict potential MoO–rice omics interaction relationships and constructed a MoO–rice multi-omics interaction network. Finally, we identified key biomolecules in the MoO–rice interaction process through functional enrichment analysis.

This subsection presents a graph autoencoder algorithm combined with the WGCNA approach to analyze the MoO–rice multi-omics relationship data. Our algorithm is motivated by two key observations. First, the WGCNA method can construct gene co-expression networks and identify regulatory genes in key modules, providing valuable insights into the MoO–rice interaction process. Second, the variational graph autoencoder (VGAE) algorithm is more suitable for inferring with low-dimensional representations from high-dimensional features of graphs, which can better capture the similarities and dependencies between nodes. The WGAEMRP algorithm consists of two main components: (1) WGCNA-based core gene identification and (2) Multi-omics relationship prediction based on an algorithm called Graph Autoencoder MoO–rice Multi-omics Relationship Prediction (GAEMRMRP). The WGCNA method integrates genomics and transcriptomic data and identifies rice core genes and MoO key sRNAs during the MoO–rice interaction process. In GAEMRMRP, we use a variational expectation maximization (EM) algorithm to alternately learn the feature inference graph autoencoder and the label propagation graph autoencoder. This approach can significantly improve prediction robustness and accuracy without manually setting feature similarities. The entire flow chart of the WGAEMRP algorithm is shown in Figure 1.

### 2.3. GAEMRMRP Model Based on GAE

**Figure 2:**
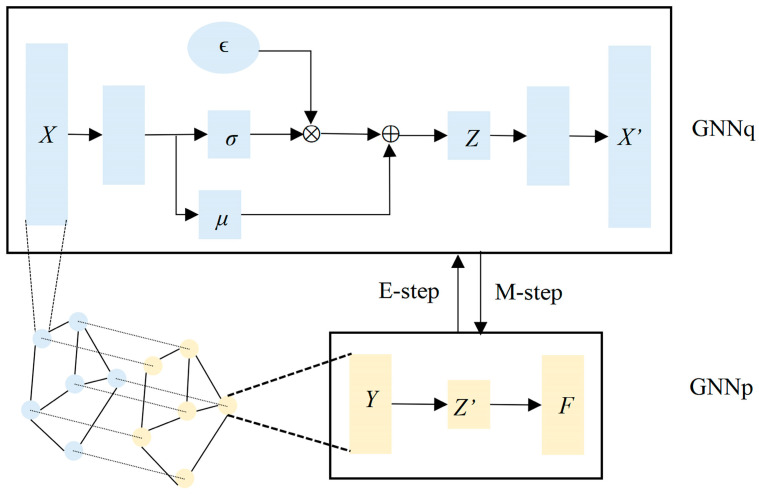
Structure of the GAEMRMRP model. The light orange GNNp in the picture is a graph autoencoder, and the light blue GNNq in the picture is a VGAE. The model is trained using the variational EM algorithm, and the E step (feature inference) and M step (label propagation) are alternately performed until convergence.

In GAEMRMRP, the feature inference network GNNq utilizes a VGAE, while the label propagation network GNNp employs a GAE. These two graph autoencoders are applied to solve the geometric matrix completion problem and capture efficient low-dimensional representations in GAEMRMRP. Additionally, GAEMRMRP adopts co-training that integrates information from the multi-omics space. The model structure can be seen in Figure 2.

Each layer of a graph autoencoder is convolutional graph layer. The formula of the *l*-th (*l* > 0) graph convolutional layer is

(1) $$H^{\left(l\right)}=\rho \left({\tilde{D}}^{-1/2}\tilde{A}{\tilde{D}}^{-1/2}H^{\left(l-1\right)}\Theta^{\left(l\right)}\right)$$

where $\tilde{A}$ is the adjacency matrix with a self-loop, i.e., $\tilde{A}=A+I$. $\tilde{D}$ is a diagonal matrix called the degree matrix,${\tilde{\;D}}_{ii}=\sum_{j}{\tilde{A}}_{ij}$, *ρ*(·) denotes nonlinear activation function, $\Theta^{\left(l\right)}$ denotes the weight of the *l*-th layer of the network, and *H*^(0)^ is the initial input feature matrix.

**Assumption** **1.** *Autoencoder GNNp with Y as the input and F as the output can obtain the optimal solution of the matrix completion problem.*

**Definition** **1.** *(manifold loss). Suppose Z and Z′ are representations of autoencoder GNNq and GNNp, respectively; then, optimizing the manifold constraint trace (FTLF) can be viewed as optimizing the following manifold loss.*

(2) $$L_{m}=\frac{1}{2}{\left\|Z-Z^{\prime }\right\|}_{F}^{2}$$

Autoencoder GNNp with the addition of the manifold loss as we defined in Definition 1 obtains the solution where geometric matrix completion introduces manifold-constrained trajectories (FTLF) into low-rank constraints.

However, to enhance the efficiency of adding the manifold loss to Equation (2), we implemented a VGAE as GNNq to capture representation *Z*. Suppose the feature matrix of the graph is *X*; the encoder learns the mean *µ* and standard deviation *σ*. The representation *Z* can be computed by applying the reparameterization trick [55], which means

(3) Z=μ+σϵ

where *ϵ* is sampled from a standard Gaussian distribution. Then, the decoder reconstructs a feature matrix *X*′.

The adjacency matrix of graph *G* can be constructed simply in this way. Firstly, sort the Euclidean distances among different feature vectors of nodes. Secondly, for each node *i*, select the 10-nearest nodes except itself. Thirdly, suppose the set of these nodes for node *i* is *N(i)*, and matrix *C* satisfies that *C_ij_* = 1 if *j*∈*N(i)*; otherwise, *C_ij_* = 0. The adjacency matrix with a self-loop of the constructed graph *G* is

(4) $$\tilde{A}=C^{T}⊙C+I$$

where ⊙ denotes the Hadamard product.

As shown in Figure 2, GNNp is a basic graph autoencoder that takes the initial label matrix *Y* as the input, the dimension of the hidden vector is 256, the output of the hidden layer is *Z*′, and the output of the decoder is prediction *F*. GNNq is a VGAE, each layer of the VGAE is a convolutional graph layer, and the dimension of the output vectors of each hidden layers in GNNq are 256.

### 2.4. WGAEMRP Algorithm Based on WGCNA and GAEMRMRP

The algorithm steps of WGAEMRP are as follows. Here is an example of the process of predicting the interaction pair between the proteome and the transcriptome.

(1) When predicting the relationship between the proteome and the transcriptome, for the features of the transcriptome data, the association with the genome data is used as the feature vector, which is *X_d_*. The feature *X_l_* of the protein sequence is calculated using Word2Vec [56], and the adjacency matrix ***Y*** is the known relationship between the proteome and the transcriptome.
(2) According to the feature *X_l_* of the protein sequence and the feature *X_d_* of the transcriptome data, the network *G_l_* and the network *G_d_* of the proteome and transcriptome are constructed, respectively.
(3) GNNql and GNNpl are applied to *G_l_*, requiring *X_l_* and *Y* as inputs, while GNNqd and GNNpd applied to *G_d_* require *X_d_* and *Y^T^* as inputs.
(4) The variational EM algorithm is used to train GNNq and GNNp alternately and collaboratively train GNNql and GNNqd. Like other VGAE, the loss function of GNNq is the sum of the reconstruction error *L_qr_* and KL divergence *L_KL_*.

(5) $$L_{q}=L_{qr}+L_{KL}$$

The loss function of GNNp is the sum of the reconstruction error and manifold loss.

(6) $$L_{p}=L_{pr}+{\gamma L}_{m}$$

Equations (5) and (6) can compute the loss from G*_l_* and G*_d_*, respectively, but it is important to integrate the information captured from multi-omics space. Therefore, we adopt co-training to train GNNql and GNNqd collaboratively.

**Definition** **2.** *(co-training loss). Suppose Z_l_ and Z_d_ are representations learned from two omics space, respectively, then co-training the loss*.

(7) $$L_{c}=\frac{1}{2}{\left\|Z_{l}Z_{d}^{T}-Y\right\|}_{F}^{2}$$

can measure the performance of the co-training.

Then, GNNql and GNNqd are trained simultaneously by optimizing the total loss of GNNq:

(8) $$L_{q}=\alpha L_{ql}+\left(1-\alpha \right)L_{qd}+\beta L_{c}$$

where *L_ql_* and *L_qd_* denote the losses of GNNql and GNNqd computed through Equation (5), respectively, and *α* ∈ (0, 1) is the weight parameter that balances the information captured from two omics space. Similarly, the total loss of GNNp is

(9) $$L_{p}=\alpha L_{pl}+\left(1-\alpha \right)L_{pd}$$

where *L_pl_* and *L_pd_* denote the losses of GNNpl and GNNpd computed through Equation (6), respectively. Then, the variational EM algorithm is implemented through optimizing $L_{q}$ and $L_{p}$ alternately. After the training procedure, GNNpl outputs *F_l_* while GNNpd outputs *F_d_*. Since both *F_l_* ∈ ℝ^m×n^ and *F_d_* ∈ ℝ^n×m^ are low ranks provided by autoencoders, and through the rank-sum inequality,

(10) $$rank\left(aF_{l}+bF_{d}^{T}\right)\leq rank\left(F_{l}\right)+rank\left(F_{d}^{T}\right),\;\forall a,b$$

(5) The final prediction result fusion is performed according to Equation (11).

(11) $$F=\alpha F_{l}+\left(1-\alpha \right)F_{d}^{T}$$

The pseudocode of the WGAEMRP algorithm is shown below Algorithm 1.
**Algorithm 1.** WGAEMRP Algorithm**Input:** Proteome features *X_l_*, transcriptome features *X_d_*, initial association matrix *Y*, and parameters α,β,γ **Output:** score matrix *F* 1: Construct graph *G_l_* and *G_d_* proteome Equation (4) from proteome features *X_l_* and transcriptome features *X_d_*, respectively 2: **repeat** 3:  $X_{l}^{\prime },\;Z_{l}\leftarrow \;GNNql\left(G_{l},X_{l}\right)$
4:  $F_{l},\;Z_{l}^{\prime }\leftarrow \;GNNpl\left(G_{l},Y\right)$
5:  $X_{d}^{\prime },\;Z_{d}\leftarrow \;GNNqd\left(G_{d},X_{d}\right)$
6:  $F_{d},\;Z_{d}^{\prime }\leftarrow \;GNNpd\left(G_{d},Y^{T}\right)$
7:  Compute *L_ql_* and *L_qd_* through Equation (5), respectively 8:  Compute *L_pl_* and *L_pd_* through Equation (6), respectively 9:  Compute the co-training loss *L_c_* through Equation (7)  // train GNNql and GNNqd collaboratively 10:  $L_{q}\leftarrow \;\alpha L_{ql}+\left(1-\alpha \right)L_{qd}+\beta L_{c}$  // Equation (8) 11:  $L_{p}\leftarrow \;\alpha L_{pl}+\left(1-\alpha \right)L_{pd}$  // Equation (9) 12:   Update the weights of GNNql, GNNpl, GNNqd, and GNNpd by optimizing $L_{q}$ and $L_{p}$ alternately  //train GNNq and GNNq alternately via the variational EM algorithm 13: **until** Convergence 14: $F\leftarrow \;{\alpha F}_{l}+\left(1-\alpha \right)F_{d}^{T}$  // Equation (11) 15: **return** *F*

## 3. Results

The forecast results of WGAEMRP have been sorted and are presented in Table 1, Table 2 and Table 3. These tables show the top 10 relationship pairs of MoO–rice proteome, transcriptome, and genome prediction scores, along with their respective prediction scores. These relational pairs will be used to construct the MoO–rice multi-omics heterogeneous interaction network, which will help explore the intrinsic relationship of biomarkers between omics. The biomolecules involved in these relationships are the master regulators that play a key role in the MoO infection of rice. Conducting biological experiments on these molecules will be crucial for the preventing and controlling of rice blast.

WGAEMRP employs AUROC and AUPR values as evaluation metrics, which are important indicators for measuring the performances of binary classification models. The model’s AUROC and AUPR values for five experiments are presented in Table 4. The results in the table demonstrate that WGAEMRP performs well in predicting the relationship between any two omics data of MoO and rice and is suitable for predicting the relationship between MoO and rice multi-omics data.

**Figure 3:**
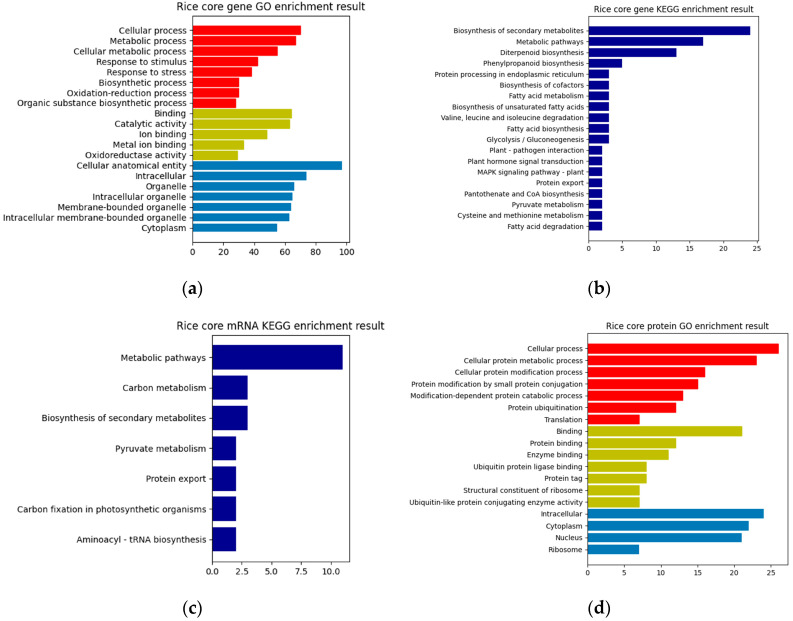
(**a**) Rice core gene GO enrichment result. (**b**) Rice core gene KEGG enrichment result. (**c**) Rice core mRNA KEGG enrichment result. (**d**) Rice core protein GO enrichment result. The red bars in (**a**) and (**d**) are Biological Processes, the yellow bars are Molecular Functions, and the blue bars are Cellular Components.

**Figure 4:**
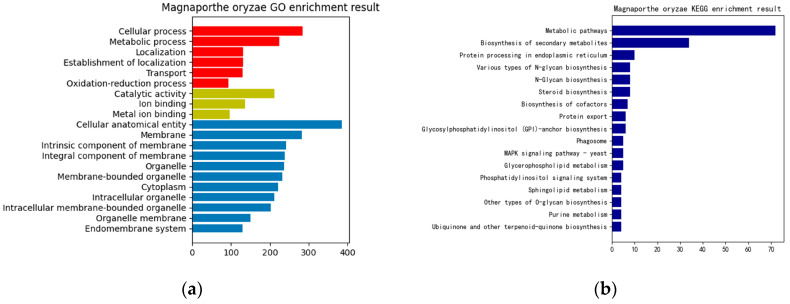
(**a**) MoO GO enrichment result. (**b**) MoO KEGG enrichment result. The red bars in (**a**) are Biological Processes, the yellow bars are Molecular Functions, and the blue bars are Cellular Components.

In this paper, a hierarchical network of omics was established horizontally based on the key genes of rice obtained from the previous analysis, the differentially expressed sRNAs of MoO, and the protein interaction pairs between MoO and rice. Then, GO enrichment and KEGG pathway enrichment analyses were performed on them. The resulting enrichment analysis chart is shown in Figure 3. Finally, the previously obtained MoO RNA interaction network, MoO mRNAs, and MoO proteins involved in the MoO–rice protein interaction network were subjected to a GO enrichment analysis and KEGG pathway enrichment analysis. The resulting enrichment analysis chart is shown in Figure 4.

Based on the enrichment results presented above, it can be concluded that the infection mechanism of MoO involves the differentially expressed rice genes, the MoO and rice mRNAs regulated by MoO sRNAs, and the interaction proteins of MoO and rice. These biomolecules are mainly related to gene expression regulation, biosynthesis, protein synthesis, processing, transportation, and metabolism and are crucial in completing the process of MoO infecting rice.

**Figure 5:**
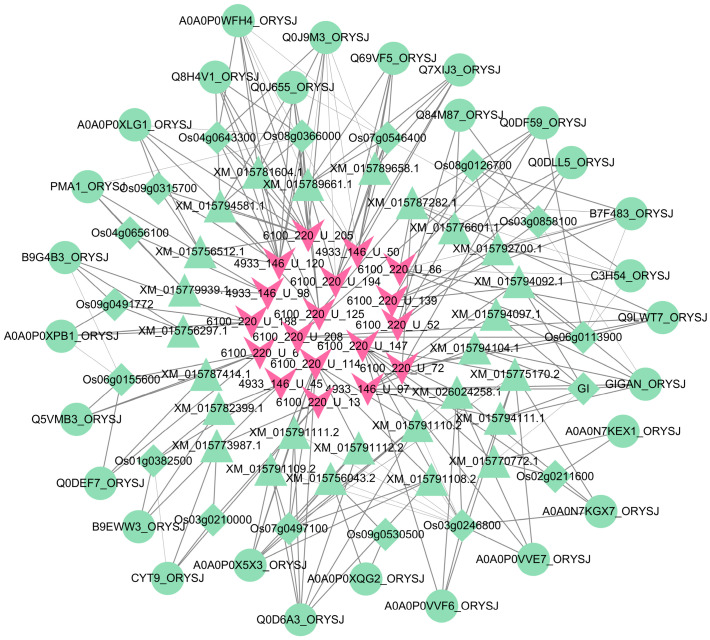
MoO–rice multi-omics hierarchical heterogeneous interaction network. The pink V-shaped nodes are the MoO sRNAs, the green diamond-shaped nodes are the rice genes, the green regular triangle nodes are the rice mRNAs, and the green circular nodes are the rice proteins.

The interactions between any two omics obtained through WGAEMRP were used to construct the MoO–rice multi-omics hierarchical heterogeneous interaction network. The network diagram is shown in Figure 5. The figure shows 18 MoO sRNAs, 17 rice genes, 26 rice mRNAs, and 28 rice proteins. A functional enrichment analysis was performed on the involved rice genes, mRNAs, and proteins. Most are related to protein processing, biosynthesis, and metabolic degradation.

Based on all the enrichment results obtained in this section, it can be concluded that, in the MoO–rice interaction mechanism, each core node of omics is primarily associated with gene expression regulation, biosynthesis, protein synthesis, processing, transportation, and metabolic processes. This indicated that the process of MoO infecting rice mainly achieves the purpose of infecting rice through the invasion mechanism of secreted proteins. At the same time, there may also be cases where MoO sRNAs play a regulatory role in the infection process. When rice defends against infection by MoO, it mainly uses related proteins in PTI and ETI. Rice sRNAs are also involved in defense responses.

## 4. Discussion

Previous studies have shown that fungal pathogens can affect the immune system of plants through the cross-kingdom regulation of sRNAs [26,27,28,29,31]. In this study, we employed a sophisticated approach, WGAEMRP, which leverages WGCNA and graph autoencoder techniques. Our objective is to uncover correlations within multi-omics data and establish a comprehensive MoO–rice multi-omics hierarchical heterogeneous network. This network allows us to identify the functional modules and enrichment pathways associated with key sRNAs participating in the interaction process.

Researchers have demonstrated that MoO sRNAs play a crucial role in regulating developmental processes, such as fungal growth and virulence, during the infection of rice by MoO. Zhang et al. developed a SVM model capable of predicting differentially expressed sRNAs of MoO, which holds significance for predicting differentially expressed sRNAs of related MoO species [53]. Our previous research involved training a prediction algorithm for cross-species interaction in proteomics, which yielded better prediction results based on the above experiments [57].

WGCNA stands out as a method adept at identifying gene sets (modules) exhibiting similar expression patterns, scrutinizing the connection between gene sets and sample phenotypes, constructing regulatory networks among gene sets, and pinpointing pivotal regulatory genes [58]. It distinguishes itself from simplistic clustering, such as Euclidean distance-based clustering, owing to its inherent biological significance. It also has the advantage of using a soft threshold that is calculated based on the weight of each gene expression, making it more suitable for practical applications than hard thresholds. Moreover, WGCNA is suitable for complex transcriptome data because of its many samples [59]. It is also suitable for studying developmental regulation in different organ/tissue types, stages, and temporal response mechanisms to biotic and abiotic stresses.

An autoencoder consists of an encoder and a decoder. The encoder obtains a high-level vector representation of the nodes, and the decoder utilizes the high-level vector representation to reconstruct the graph structure. The graph autoencoder uses the graph convolutional network as the encoder. It takes the node features and adjacency matrix as the input to obtain the node’s latent representations (or embedding). The graph autoencoder then uses the inner product as a decoder to reconstruct the original graph. The graph autoencoder can be used to generate latent vectors like autoencoders and can also be used for link prediction.

In contrast, variational autoencoders (VAE) utilize probabilistic methods to describe distinctions in the latent space and constitute a generative model within the realm of unsupervised learning. VAE’s encoder and decoder output probability density distributions of variables subject to parameter constraints, while the autoencoder encodes a specific value. Migrate VAE to the graph field, encode the known graph through the graph convolution layer, learn the distribution of the node vector representations, sample the node vector representations in the distribution, and then decode and reconstruct. After VGAE obtains the graph node encoding, it calculates the probability of edges between nodes pairwise and reconstructs the graph based on this. VGAE can be used for link prediction.

This paper integrates the genomics, transcriptomic, and proteomic data of MoO and rice to propose the WGAEMRP model based on WGCNA and graph autoencoder. The model predicts associations among MoO–rice multi-omics data. Compared to previous methods, the efficiency and accuracy of this approach have greatly improved. Table 5 presents a comparison of the three groups of MoO–rice interaction mechanism research, including this paper.

Rice expressing the blast resistance (R) gene, termed the *Pi* gene, is resistant to races or strains of the rice blast fungus *M. oryzae Oryzae* pathotype that expresses *AVR-Pi* in a gene-for-gene relationship. Although R genes confer strong resistance responses, with the exception of *Pi9*, none of these R genes are persistent or confer broad-spectrum resistance [60]. The collapse of resistance is caused by the rapid adaptation of the pathogen, i.e., the evolution of new races that overcome the introduced resistance genes. The rapid evolution of new races is attributed to the rapid loss of function of avirulent effector genes that correspond to resistance genes in a gene-for-gene manner. The loss of avirulent function may be due to point mutations, including repeat-induced point mutations, the insertion of transposable elements, or deletion of the entire gene via the process of asexual reproduction. When varieties containing the corresponding resistance genes are removed from the field, fungal populations often reacquire the expelled avirulence genes [61]. Therefore, it is of great significance for researchers to conduct research on R genes and AVR genes for the prevention and treatment of rice blast.

Using the data analysis method proposed in this study, we identified 18 key MoO sRNAs. We hypothesize that some MoO sRNAs can regulate the process of infecting rice by targeting rice mRNAs for rice RNA silencing. Some MoO sRNAs may increase the number of certain proteins in MoO, thereby infecting rice by secreting proteins. We used the tool psRNATarget to target the predicted MoO sRNAs to MoO avirulence genes and rice resistance genes. A total of 11 MoO sRNAs can be targeted to MoO avirulence genes or rice resistance genes (Table 6), which, to a certain extent, verifies the reliability of our developed method.

## 5. Conclusions

The experimental results demonstrate that WGAEMRP’s tremendous potential for analyzing complex regulatory mechanisms across species outperforms the previous methods. Integrating genomics and transcriptomic data through WGCNA allows to identify core rice genes and key MoO sRNAs during infection. Additionally, WGAEMRP utilizes the variational EM algorithm to alternately learn the feature inference graph autoencoder and the label propagation graph autoencoder. This approach enhances WGAEMRP’s ability to capture low-dimensional representations from high-dimensional features, improving the accuracy and robustness of predicting unknown associations between different omics data.

To optimize the WGAEMRP algorithm, we plan to consider some technical solutions in the future. For instance, the model can be further developed to analyze the three omics data together and directly predict the relationship between them, which will efficiently construct the MoO–rice multi-omics heterogeneous interaction network and identify key biomolecules. Additionally, this article only studies the infection mechanism of MoO; it ignores the analysis of the defense mechanism of rice. In the future, we will comprehensively improve the research on the mutual regulatory process of MoO and rice. Finally, while WGAEMRP currently focuses on MoO infecting rice, we aim to extend its application to all fungus–plant interaction fields in the future, such as Botrytis cinerea infecting tomatoes and Phytophthora infestans infecting potatoes.

## Figures and Tables

**Table 1:** The top 10 relationship pairs of MoO–rice proteome and transcriptome prediction scores and their prediction scores.

Protein	sRNA/mRNA	Score
Q0J9M3_ORYSJ	XM_015789661.1	0.256037617
Q0J9M3_ORYSJ	4933_146_U_50	0.254859433
Q0J9M3_ORYSJ	XM_015789658.1	0.253295534
A0A0P0WFH4_ORYSJ	XM_015789661.1	0.251212673
A0A0P0WFH4_ORYSJ	4933_146_U_50	0.251002283
C3H54_ORYSJ	XM_015787282.1	0.25043423
B7F483_ORYSJ	XM_015787282.1	0.250195788
C3H54_ORYSJ	6100_220_U_86	0.249845138
Q0J9M3_ORYSJ	6100_220_U_194	0.249648774
B7F483_ORYSJ	6100_220_U_86	0.249606696

**Table 2:** The top 10 relationship pairs of MoO–rice proteome and genome prediction scores and their prediction scores.

Protein	Gene	Score
C3H54_ORYSJ	Os06g0113900	0.22856142
B7F483_ORYSJ	Os06g0113900	0.228370044
A0A0P0XLG1_ORYSJ	Os08g0366000	0.215526588
Q0J655_ORYSJ	Os03g0858100	0.210768768
B9G4B3_ORYSJ	Os06g0155600	0.199169256
A0A0P0XPB1_ORYSJ	Os06g0155600	0.194762292
CYT9_ORYSJ	Os01g0382500	0.190769976
GIGAN_ORYSJ	Os03g0858100	0.18874458
A0A0P0WFH4_ORYSJ	Os07g0546400	0.184396092
PMA1_ORYSJ	Os08g0366000	0.182716236

**Table 3:** The top 10 relationship pairs of MoO–rice proteome and transcriptome prediction scores and their prediction scores.

sRNA/mRNA	Gene	Score
XM_015775170.2	Os07g0497100	0.392323263
XM_015756043.2	Os07g0497100	0.389914638
XM_026024258.1	Os07g0497100	0.273902412
XM_015791110.2	Os03g0246800	0.221535693
XM_015791108.2	Os03g0246800	0.188026902
XM_015756043.2	Os03g0246800	0.187596561
XM_015791111.2	Os03g0246800	0.187570869
XM_015791112.2	Os03g0246800	0.187570869
XM_015791109.2	Os03g0246800	0.187333218
XM_015770772.1	GI	0.182458161

**Table 4:** AUROC and AUPR values for 5 experiments performed by WGAEMRP.

**Proteome–Transcriptome**							
Time	1	2	3	4	5	Mean	Stdev
AUROC	0.9432	0.9459	0.9471	0.9498	0.9642	0.9500	0.0083
AUPR	0.3901	0.6939	0.7013	0.3917	0.6646	0.5683	0.1625
**Proteome–Genome**							
Time	1	2	3	4	5	Mean	Stdev
AUROC	0.8955	0.8897	0.8923	0.8974	0.9150	0.8980	0.0100
AUPR	0.2514	0.5146	0.4913	0.2259	0.5316	0.4030	0.1509
**Transcriptome–Genome**							
Time	1	2	3	4	5	Mean	Stdev
AUROC	0.9017	0.9088	0.9042	0.8648	0.8999	0.6423	0.0177
AUPR	0.3091	0.6294	0.4848	0.5555	0.6423	0.5242	0.1358

**Table 5:** Comparison of the research methods for the interaction mechanism of MoO–rice.

Algorithm Name	Omics	Basic Algorithms	Results	Advantages	Disadvantages
/	Genome and transcriptome	SVM	Key sRNA of MoO	Screened out differentially expressed sRNAs of MoO	Lacking the support of proteomic data
/	Genome, transcriptome and proteome	RNN	Cross-species protein interaction network	Analysis of cross-species regulatory mechanism	Model prediction only for proteomic data
WGAEMRP	Genome, transcriptome and proteome	WGCNA and graph autoencoder	Multi-omics heterogeneous interaction network	Predicted for multi-omics relationship data	The model failed to predict the relationship between the three omics directly

**Table 6:** Eleven key sRNAs of MoO that can target MoO avirulence genes or rice resistance genes.

sRNA	Target_Accession Number	Target_Name	Target_Organism
4933_146_U_50	AJ704622.1; KP985760.1; MH807580.1	ACE1; Pi50; Pizh	Pyricularia grisea; Oryza sativa Japonica Group
6100_220_U_147	EU837058.1; DQ285630.1; MH807580.1	AvrPiz-t; Pi9; Pizh	Pyricularia grisea; Oryza sativa Indica Group; Oryza sativa Japonica Group
6100_220_U_188	EU837058.1; DQ285630.1; MH807580.1	AvrPiz-t; Pi9; Pizh	Pyricularia grisea; Oryza sativa Indica Group; Oryza sativa Japonica Group
6100_220_U_194	AJ704622.1; KP985760.1; MH807580.1	ACE1; Pi50; Pizh	Pyricularia grisea; Oryza sativa Japonica Group
6100_220_U_208	KM522920.1	IUG9	Pyricularia oryzae
6100_220_U_86	KM522920.1	IUG9	Pyricularia oryzae
4933_146_U_97	KP985760.1; MH807580.1	Pi50; Pizh	Oryza sativa Japonica Group
4933_146_U_98	KP985760.1; MH807580.1	Pi50; Pizh	Oryza sativa Japonica Group
6100_220_U_139	MH807580.1	Pizh	Oryza sativa Japonica Group
6100_220_U_52	DQ285630.1; KP985760.1; MH807580.1	Pi9; Pi50; Pizh	Oryza sativa Indica Group; Oryza sativa Japonica Group
6100_220_U_72	HM048900.1	Pik	Oryza sativa Japonica Group

## Data Availability

Publicly available datasets were analyzed in this study. This data can be found here: https://www.ncbi.nlm.nih.gov/geo/query/acc.cgi?acc=GSM2049367:; https://www.ncbi.nlm.nih.gov/geo/query/acc.cgi?acc=GSE43277; https://www.ncbi.nlm.nih.gov/geo/query/acc.cgi?acc=GSE110088; https://www.ncbi.nlm.nih.gov/geo/query/acc.cgi?acc=GPL2025; https://rapdb.dna.affrc.go.jp/download/irgsp1.html; https://www.ncbi.nlm.nih.gov/Traces/study/?acc=SRX214117; https://www.ncbi.nlm.nih.gov/Traces/study/?acc=SRX214123; https://www.ncbi.nlm.nih.gov/geo/query/acc.cgi?acc=GSM973470; https://www.ncbi.nlm.nih.gov/geo/query/acc.cgi?acc=GSM973471; https://www.ncbi.nlm.nih.gov/geo/query/acc.cgi?acc=GSM2049367; https://www.ncbi.nlm.nih.gov/Traces/wgs/AACU03?val=AACU03.1; https://www.ncbi.nlm.nih.gov/Traces/wgs/AACU03?val=LVCG01.1; https://hpidb.igbb.msstate.edu/about.html#stats (accessed on 5 April 2022).
